# Supporting Caregiver Well-Being Through a Virtual Art Exhibit: Pilot Study Qualitative Findings

**DOI:** 10.1093/geroni/igaf122.3661

**Published:** 2025-12-31

**Authors:** Charlotte Weiss, Katherine Cotter, Morgan Delizia, Connie Ulrich, Karen Hirschman

**Affiliations:** University of Pennsylvania, Philadelphia, Pennsylvania, United States; University of Pennsylvania, Philadelphia, Pennsylvania, United States; University of Pennsylvania, Philadelphia, Pennsylvania, United States; University of Pennsylvania School of Nursing - Philadelphia, PA, philadelphia, Pennsylvania, United States; University of Pennsylvania, Philadelphia, Pennsylvania, United States

## Abstract

The growing number of recently hospitalized older adults with multiple chronic conditions (MCCs) increasingly relies on family caregivers (FCGs) for essential support. While FCG involvement is critical, the caregiving role often has a negative impact on their social, emotional, and spiritual well-being. Emerging evidence suggests that engaging with visual art, including museum visits, can enhance well-being across these domains. However, little is known about the use of virtual art exhibits to support FCGs of older adults with MCCs. This exploratory, multi-methods feasibility study aimed to develop and pilot a virtual art museum experience for FCGs of recently hospitalized older adults with MCCs. We first conducted semi-structured interviews with five FCGs to explore their perceptions of virtual art engagement, including potential benefits and barriers. Next, we used the insights from these interviews to inform the curation of a 14-day virtual art exhibit using images from Google Arts & Culture. The exhibit was piloted with a convenience sample of FCGs (n = 15). A daily diary approach was used to explore the associations between daily engagement and perceived stress, social connectedness, emotional well-being, and spirituality. The qualitative findings gathered from the daily diary entries suggest that daily engagement with a virtual art exhibit is both acceptable and accessible for FCGs. The themes identified will be highlighted. Virtual art engagement may offer a scalable, low-cost behavioral intervention to support the holistic well-being of family caregivers managing the complex demands of caregiving for older adults with MCCs.

